# Supplementary material for: WordCommentsAnalyzer: A windows software tool for qualitative research
Source: F1000Res. 2018 Sep 4;7:536. Originally published 2018 May 3. [Version 2] doi: 10.12688/f1000research.14819.2 (PMC6137411; doi:10.12688/f1000research.14819.2)
Supplement: Supplementary file 3 [file f1000research-7-17614-s0002.tgz › b2a79496-4ada-47c4-9682-a99ce867836d.zip!/Eight Iranian Health Journals/The Journal of Toloo-e-Behdasht_21.docx]

© The Journal of Toloo-e-Behdasht. This work is licensed under CC BY 4.0 (https://creativecommons.org/licenses/by/4.0/).

Volume & Issue: دوره 13 - شماره 6

Title: بررسی میزان جذب فومونیزین B1 توسط نانوذرات سلولز اصلاح شده با پلی لیزین در حضور مواد غذایی مختلف

http://tbj.ssu.ac.ir/article-1-1369-fa.html

مقدمه: فومونیزین B1 مایکوتوکسینی است که باعث ایجاد صدمات عصبی، سرطان مری ، بیماری های کبد و کلیه می گردد. هدف از این مطالعه بررسی توانایی نانوذرات سلولز اصلاح شده با پلی لیزین برای حذف سم فومونیزین B1 در حضور مواد غذایی مختلف بود. روش‌ بررسی: نخست نانوذرات سلولز با روش هیدرولیز اسیدی سنتز و سپس با اسید سیتریک اصلاح و نهایتا با کمک کراس لینکر به پلی لیزین متصل گردید. سپس چهار نوع ماده غذایی شامل آرد گندم، آرد برنج، آرد ذرت و میوه خیار تهیه گردید و به هریک بطور جداگانه غلظت های مختلف از محلول فومونیزین اضافه شد. سپس به همه لوله ها نانوذرات اصلاح شده با پلی لیزین با غلظت 2000 میکروگرم بر میلی لیتر اضافه و بعد از انکوباسیون غلظت دقیق سم در هر لوله با روش کروماتوگرافی مایع با کارآیی بالا بدست آمده و نهایتا درصد جذب در هر لوله در مقایسه با کنترل محاسبه شد. یافته ها: این مطالعه آشکار ساخت که هر چند این نانوذرات در حضور هر چهار نوع ماده غذایی قادر به جذب سم فومونیزین هستند، ولی بیشترین میزان جذب (95 درصد) در خیار دیده شد. همچنین نتایج آزمون ها نشان می دهد که درصد جذب با غلظت اولیه سم فومونیزین موجود در ماده غذایی ارتباط مستقیم دارد. نتیجه گیری: در این مطالعه نانوذرات اصلاح شده با پلی لیزین بعنوان جاذب سم فومونیزین B1 برای اولین بار معرفی گردید. این نانوذرات توانستند منجر به حذف سم فومونیزین B1 در حضور مواد غذایی مختلف شوند.

Title: تحلیلی بر روش‌های جدید حذف نیترات از منابع آب

http://tbj.ssu.ac.ir/article-1-1370-fa.html

مقدمه: نیترات به عنوان یک آلاینده مقدم در منابع آب شناخته شده است. این آلاینده باعث ایجاد مشکلات بهداشتی مانند سندرم بچه آبی در نوزادان و ایجاد سرطان در دستگاه گوارش می‌گردد. بدلیل عدم وجود مطالعه ای جامع در زمینه روش های جدید حذف نیترات از منابع آب در ایران لزوم معرفی فن آوری های حذف نیترات، ویژگی ها، راندمان و نحوه تاثیر آن‌ها بر حذف نیترات و معایب و مزایای هر کدام محسوس می باشد. روش بررسی: در این بررسی مطالعات مرتبط صورت گرفته در دهه اخیر از بانکهای اطلاعاتی Google Scholar, Pub med, Elsevier, Scopus و SID با کلید واژه های نیترات، منابع آب، روشهای فیزیکوشیمایی و بیولوژیکی به صورت فارسی و انگلیسی جستجو شد. یافته ها: در بین روش های معمول حذف نیترات صرفه نظر از مسائل اقتصادی به ترتیب کارایی روش تبادل یونی، اسمز معکوس، روش های احیای الکتریکی، دنیتریفیکاسیون و جذب در حذف نیترات موثر بوده اند. نتیجه گیری: با توجه به اینکه روش های مذکور استاندارد سازمان جهانی بهداشت در زمینه یون نیترات در آب آشامیدنی را فراهم می آورد انتخاب یک روش بهینه منوط به در نظر گرفتن جنبه های اقتصادی، فنی و مهندسی، منطقه ای و شرایط آب و هوایی می‌باشد.

Title: بررسی رضایت بیماران از خدمات پزشکان خانواده مراکز بهداشتی درمانی روستایی شهرستان سبزوار با ابزار EUROPEP -1391

http://tbj.ssu.ac.ir/article-1-1371-fa.html

مقدمه: طرح پزشک خانواده یکی از تحولات اساسی نظام سلامت ایران است و رضایت دریافت کنندگان خدمات یکی از مهمترین مسائلی است که باید در این طرح مورد توجه قرار گیرد، لذا پژوهش حاضر با هدف بررسی رضایت بیماران از خدمات پزشکان خانواده مراکز بهداشتی درمانی روستایی شهرستان سبزوار در سال 1391 انجام شده است. روش بررسی: در این مطالعه توصیفی- تحلیلی، جامعه پژوهش را مردم تحت پوشش مراکز بهداشتی درمانی روستایی سبزوار تشکیل می دادند که 266 نفر از آنان به صورت خوشه ای و تصادفی انتخاب شدند. داده ها به صورت میدانی و با استفاده از پرسشنامه‌ گردآوری شده و با استفاده از آمار توصیفی (فراوانی، درصد، میانگین، انحراف معیار)، آزمون های همبستگی (05/0P≤) و نرم افزار 11 SPSS تحلیل شد. یافته ها: نتایج مطالعه نشان داد 2/40% جمعیت از خدمات ارائه شده توسط پزشک خانواده رضایت عالی، 6/43% رضایت خوب، 8/15% رضایت متوسط و 4/0% رضایت ضعیف داشتند. میزان رضایت به تفکیک در بخش های خوشاب، داورزن و روداب به ترتیب 2/40% ، 7/29% و 1/30% بود. بین رضایتمندی با سن، جنسیت، سطح تحصیلات، علت مراجعه و وضعیت تاهل تفاوت آماری معناداری مشاهده نشد. نتیجه گیری: در مجموع، برنامه پزشک خانواده در شهرستان سبزوار موفق بوده است و با اصلاح برخی نقایص سیستم بر اساس نظرات گیرندگان خدمت می توان رضایت مندی بالاتری از برنامه را نیز حاصل کرد.

Title: بررسی کارایی میکروارگانیسم های خالص سازی شده از خاکهای حاوی گازوئیل در تولید بیوسورفکتانت

http://tbj.ssu.ac.ir/article-1-1372-fa.html

مقدمه: آلودگی خاک با گازوئیل منجر به ضایعات شدید زیست محیطی در اکوسیستم می گردد. لذا محققان در سراسر دنیا روش های گوناگونی را برای حذف گازوئیل از خاک مورد بررسی قرار داده اند. هدف از انجام این مطالعه استخراج مواد بیوسورفکتانت به منظور کاربرد در تصفیه خاک های آلوده به گازوئیل بود. روش بررسی: در این مطالعه از چهار منطقه که مدتها در تماس با ترکیبات گازوئیلی بوده اند نمونه برداری شد. نمونه ها برای یافتن میکروارگانیسم هایی که قابلیت تولید بیوسورفکتانت و مصرف گازوئیل را داشته باشند مورد بررسی قرار گرفت. در مرحله بعد توانایی میکروارگانیسم ها در تولید بیوسورفکتانت توسط آزمون امولسیون سازی (E24) مورد بررسی قرار گرفت. در نهایت بیوسورفکتانت محلول در محیط کشت مغذی استخراج و اندازه گیری شد. یافته ها: در این مطالعه چهار گونه باکتری خالص سازی شد. تمام باکتریهای جدا شده گرم منفی بودند. بیشترین شاخص امولسیون سازی معادل 66 درصد بود که از باکتریهای جدا شده از خاک پمپ بنزین بدست آمد. در این مطالعه به ازای هر یک لیتر محیط کشت معدنی 1/1 گرم بیوسورفکتانت خالص سازی گردید. نتیجه گیری: نتایج این مطالعه نشان داد که برخی باکتریها قادر به تولید مقدار کافی بیوسورفکتانت هستند که قابلیت کاربرد در فرایند شستشوی خاک آ لوده به گازوئیل را دارا می باشند.

Title: ارزیابی ریسک خطاهای انسانی و ارائه اقدامات اصلاحی در پروسه کنترل نیروگاه سیکل ترکیبی با استفاده از روش رویکرد سیستماتیک پیش بینی و کاهش خطاهای انسانی SHERPA

http://tbj.ssu.ac.ir/article-1-1373-fa.html

مقدمه: بروز خطای انسانی در بسیاری از محیط های شغلی نظیر نیروگاه ها، صنایع نظامی، شیمیایی و صنایع هسته ای امری اجتناب ناپذیر می باشد که می تواند به یک فاجعه تبدیل شده و خسارات جبران ناپذیری را به همراه داشته باشد. به همین دلیل این پژوهش با هدف شناسایی و پیش بینی خطای انسانی و ارائه راهکارهای کنترلی برای کاهش خطای انسانی با استفاده از تکنیک SHERPA در نیروگاه سیکل ترکیبی انجام گرفت. روش بررسی: مطالعه حاضر یک مطالعه توصیفی مقطعی می باشد که در اتاق کنترل نیروگاه سیکل ترکیبی یزد اجرا شده است. برای تکمیل برگه ها ی SHERPA از روش مشاهده، مصاحبه با متخصصین فرآیند و اپراتورهای اتاق کنترل و آنالیز سلسله مراتبی وظیفهHTA که مقدمه ای بر انجام آنالیز خطای انسانی می باشد استفاده شده است.رویکرد پیش بینی و کاهش خطای انسانی برای شناسایی خطاهای انسانی بالقوه در هر یک از وظایف اتاق کنترل استفاده گردید و راهکارهای کنترلی ارائه شد. یافته ها: تجزیه وتحلیل برگه های SHERPA نشان داد که تعداد کل خطاهای انسانی شناسایی شده در وظایف شغلی 115 خطا می باشد که از این تعداد 08/38% خطاها از نوع عملکردی, 42/39% بازدیدی،61/8% بازیابی و 89/13% خطا مربوط به تبادل اطلاعات می باشد. نتیجه‌گیری: بارزترین خطایی که در اتاق کنترل واحد بخار اتفاق می افتد مربوط به خطای عملکردی و بازدید می باشد.لذا آموزش کارکنان و تدوین و ترجمه دستورالعملهای کاری جهت نظارت و بازرسی دقیق اپراتورها و انجام اقدام مناسب در صورت بروز نقص در واحد، در اولویت اقدامات اصلاحی قرار گرفت.

Title: بهداشت فردی و عوامل تعیین کننده آن در نیروهای نظامی

http://tbj.ssu.ac.ir/article-1-1374-fa.html

مقدمه: بهداشت فردی یکی از مهمترین ابزارهای پیشگیری در حوزه سلامت همگانی است و نیروهای نظامی ممکن است در معرض تهدیدهای ناشی از عدم رعایت اصول بهداشت فردی باشند. هدف از این مطالعه ارزیابی وضعیت بهداشت فردی در میان نیروهای نظامی و شناخت عوامل تعیین کننده آن و موانع احتمالی مربوطه می باشد. روش بررسی: این یک مطالعه توصیفی مقطعی است که در بین 502 نفر از نیروهای نظامی از 10 مرکز نظامی واقع در سراسر ایران صورت گرفته است. این مراکز بصورت تصادفی انتخاب شدند. ابزار گردآوری داده ها پرسشنامه خودگزارشی می باشد که از 5 قسمت شامل اطلاعات دموگرافیک، آگاهی ، نگرش و عملکرد در زمینه بهداشت فردی و یک بخش در مورد موانع احتمالی تشکیل شده است. روابط بین متغیرهای دموگرافیک، آگاهی و نگرش به عنوان متغیرهای مستقل با رفتار بهداشت فردی به عنوان متغیر وابسته مورد بررسی قرار گرفتند و موانع و مشکلات مطرح شده رتبه بندی شدند. داده ها با استفاده از آزمونهای تی مستقل و همبستگی پیرسون و رگرسیون خطی مورد ارزیابی قرار گرفتند. از نرم افزار SPSS نسخه 20 برای تجزیه و تحلیل داده ها استفاده گردید. یافته ها: میانگین سنی شرکت کنندگان 83/6± 04/33 سال و بیش از 84 درصد متاهل بودند. مهم ترین منبع کسب اطلاعات در میان شرکت کنندگان، رادیو و تلویزیون گزارش گردید (3/72). سطح آگاهی (5/71%)، نگرش (7/63%) و عملکرد (8/61%) اکثر شرکت کنندگان در سطح مطلوب برآورد گردید. متغیرهای شغل، آگاهی و نگرش دارای رابطه معنی دار یا متغیر رفتار بهداشت فردی شناخته شدند (05/0 p<). عوامل تعیین کننده بهداشت فردی عبارت بودند از : درجه مصوب، مدت خدمت، شغل، آگاهی و نگرش. نتیجه گیری: با توجه به سطح آگاهی ، نگرش و عملکرد افراد در حوزه بهداشت فردی ، توصیه می شود برنامه ریزی و مداخلات آموزش مناسب در راستای بهبود این متغیرها در میان نیروهای نظامی صورت گیرد. انجام مطالعات بیشتر در بین نیروهای مختلف نظامی و دیگر اقشار جامعه به منظور شناخت هرچه بیشتر عوامل موثر در تعیین رفتار بهداشت فردی پیشنهاد می گردد.

Title: بررسی دیدگاه مردم و کارشناسان بهداشتی در مورد نقش و عملکرد رسانه‌های محلی استان یزد در برنامه‌های سلامت عمومی

http://tbj.ssu.ac.ir/article-1-1375-fa.html

مقدمه: از دیر باز اﻃﻼع‌رﺳﺎﻧﻲ دﻗﻴﻖ و ﺳﺮﻳﻊ و اراﺋﺔ آﻣﻮزشﻫـﺎی اﺛـﺮﺑﺨﺶ در زﻣﻴﻨـﻪ ﻣـﺴﺎﺋﻞ مرﺗﺒﻂ ﺑﺎ ﺳﻼﻣﺖ ﺟﺴﻤﻲ و رواﻧﻲ، وﻇﻴﻔﺔ رﺳﺎﻧﻪ ﺷﻤﺮده شده است. ولی ظاهراً رسانه‌ها بویژه رسانه‌های محلی از این وظیفه خود غافل شده‌اند. پژوهش به بررسی نگرش مردم عادی و کارشناسان بهداشت در زمینه نقش و اهمیت رسانه‌ها در حوزه سلامت و همچنین عملکرد آن‌ها در این زمینه پرداخته است. روش بررسی: این مطالعه توصیفی-مقطعی است. جامعه مورد پژوهش، کارشناسان بهداشت و مردم عادی شهر یزد بودند که در دو نمونه 211 نفری (کارشناسان) و 246 نفری (مردم عادی) وارد مطالعه شدند. ابزار گردآوری اطلاعات، پرسشنامه محقق ساخته بوده است. پرسشنامه حاوی 18 سؤال باز و بسته بود. جهت تجزیه و تحلیل داده‌ها از نرم‌افزار آماری SPSS استفاده شد و از آمار توصیفی و آزمون‌های کای‌اسکویر و یومن‌ویتنی در سطح 05/0 استفاده شد. یافته‌ها: کلیه شرکت‌کنندگان با نقش رسانه‌های محلی در زمینه موضوعات مختلف با بهداشت موافق بودند و از این حیث تفاوت معنی‌داری بین این دو گروه وجود نداشت. تنها در زمینه اصلاح تصورات ذهنی نادرست افراد جامعه در زمینه بهداشت، کارشناسان نقش رسانه‌های محلی را نسبت به مردم عادی بیشتر ارزیابی کردند (038/0 = P). بین نگرش مردم عادی و کارشناسان در مورد عملکرد رسانه‌های محلی استان یزد در زمینه جلب مشارکت همگانی تفاوت معنی‌داری وجود ندارد (355/0 = P). نتیجه‌گیری: کلیه شرکت‌کنندگان معتقد بودند که رسانه‌ها می‌توانند نقش مهمی در تحقق مشارکت همگانی در حوزه سلامت ایفا نمایند ولی هر دو گروه، عملکرد فعلی رسانه‌های محلی را ضعیف ارزیابی نمودند که نشان می‌دهد باید در این زمینه تدابیری اندیشیده شود.

Title: مدل های خاص تصمیم گیری چند معیاره درمداخلات آموزش بهداشت و ارتقاسلامت:مقاله مروری

http://tbj.ssu.ac.ir/article-1-1376-fa.html

مقدمه: نیازهای مطرح شده ، تصمیمات در آموزش بهداشت و ارتقا سلامت معمولا به طور همزمان تحت تاثیر عوامل متعدد قرار می گیرد،بدین معنی که پژوهشگران با محیط تصمیم گیری چند معیاره مواجه می شوند که همین امر طراحی و انتخاب گزینه صحیح را با حساسیت بیشتری روبرو می سازد. با توجه به اهمیت اصول علمی در تصمیم گیری مربوط به مداخلات مطالعه حاضر به معرفی و تبیین مدلهای تصمیم گیری چند معیاره در آموزش بهداشت و ارتقا سلامت می پردازد. روش بررسی:مقاله حاضر با بررسی متون خارجی منتشر شده درپایگاههای اطلاعاتی Web of Sciences Science Direct Medline , Google Scholar، متون فارسی منتشر شده ، بررسی کتب مربوطه و پایان نامه های فارسی به خصوص در زمینه آموزش بهداشت و ارتقا سلامت و با استفاده از کلید واژه مرتبط به بررسی مدلهای خاص تصمیم گیری چند معیاره در آموزش بهداشت و ارتقا سلامت پرداخت. یافته ها: بررسی پژوهش ها و مقالات آموزش بهداشت و ارتقا سلامت به خصوص در ایران نشان می دهد که اگرچه تکنیکهای تصمیم گیری نظیر روش دلفی، گروههای اسمی و بارش افکار و سایر رویکردها مورد استفاده قرار گرفته ولی روشهای تصمیم گیری چند معیاره که دارای پشتوانه تئوریکی ریاضی و صحت پیش بینی نتایج هستند مورد غفلت قرار گرفته است . نتیجه گیری : به کارگیری مدلهای تصمیم گیری چند معیاره در حیطه تصمیمات ارتقا سلامت که نوعا طیف وسیعی از ملاحظات وعوامل مختلف را همزمان علاوه بر ارزیابی اقتصادی در آموزش بهداشت و ارتقا سلامت مد نظر قرار می دهد ضروری است.

Title: رابطه سرسختی روان شناختی با احساس غربت دانشجویان غیر بومی دانشگاه سیستان و بلوچستان

http://tbj.ssu.ac.ir/article-1-1377-fa.html

مقدمه: از مشکلات دانشجویان غیر بومی می توان به مسئله احساس غربت آنان اشاره کرد و یکی از عواملی که می تواند با این احساس رابطه داشته باشد سرسختی روان شناختی است. بدین منظور مطالعه حاضر با هدف بررسی رابطه سرسختی روان شناختی با احساس غربت دانشجویان غیر بومی دانشگاه سیستان و بلوچستان انجام گرفت. روش بررسی: مطالعه حاضر از نوع توصیفی- همبستگی و جمعیت مورد مطالعه آن دانشجویان غیر بومی ساکن در خوابگاه های دانشجویی دانشگاه سیستان و بلوچستان در سال تحصیلی 92-1391 بودند.که به شیوه نمونه گیری تصادفی تعداد 338 نفر از آنان در دی ماه مورد مطالعه قرار گرفتند. ابزار جمع آوری اطلاعات دو پرسشنامه احساس غربت اژه ای و دیگران (1387) و مقیاس سرسختی روان شناختی کوباسا (1990) بود. برای تجزیه و تحلیل داده ها از آزمون ضریب همبستگی و رگرسیون چندگانه با استفاده از نرم افزار SPSS17استفاده شد. یافته ها: یافته ها نشان داد دانشجویان دختر در مقایسه با دانشجویان پسر احساس غربت بیش تر (01/0>P) و سرسختی روان شناختی کمتر را تجربه می کنند(05/0>P). همچنین بین سرسختی روان شناختی (و خرده مقیاس های سه گانه آن) و احساس غربت دانشجویان رابطه منفی و معنی داری مشاهده شد (01/0> P،798/0- =R ) و نتایج رگرسیون چندگانه نشان داد که از خرده مقیاس های سه گانه سرسختی روان شناختی خرده مقیاس تعهد قابلیت تبیین و پیش بینی احساس غربت دانشجویان را دارد (01/0> P، 661/4- =T ). نتیجه گیری: هرچه دانشجویان جدیدالورودِ ساکن خوابگاه، از نظر ویژگی شخصیتی سرسختی روان شناختی در سطح بالاتری باشند احساس غربت کمتری را تجربه می کنند.

Title: بررسی همبستگی تعداد دندانهای از دست رفته به عنوان شاخصی از سلامت دهان، با ظرفیت آنتی اکسیدانی کل بزاق کامل غیرتحریکی

http://tbj.ssu.ac.ir/article-1-1608-fa.html

چکیده مقدمه: از دست رفتن سلامت دهان نقش بسزایی در افت کیفیت زندگی افراد جامعه ایفا می کند. نگرانی های اخیر از تاثیر سوء بهداشت دهانی ضعیف و از دست دادن دندانها بر خطر ابتلا به بدخیمی های سر و گردن، حوزه جدیدی در تحقیقات بهداشت دهان و دندان گشوده است. مطالعه حاضر با هدف بررسی ارتباط ظرفیت آنتی اکسیدانی کل بزاق کامل غیر تحریکی با تعداد دندانهای از دست رفته انجام گرفت. روش بررسی: در این مطالعه از بین مراجعین به کلینیک دانشکده دندانپزشکی شهید صدوقی یزد 90 نفر داوطلب با حدود سنی 30 تا 49 سال شرکت نمودند. تعداد دندانهای از دست رفته در این افراد که فاقد هرگونه بیماری سیستمیک، ضایعه دهانی، و سابقه مصرف دخانیات بودند، ثبت شده و نمونه بزاق غیرتحرکی از آنها جمع آوری گردید. ظرفیت آنتی اکسیدانی کل در نمونه ها تعیین و رابطه آن با تعداد دندانهای از دست رفته مورد تحلیل آماری قرار گرفت. یافته ها: نتایج آزمون ANOVA نشان داد که میانگین دندان های از دست رفته 59/1±37/2 و میانگین آنتی اکسیدان توتال بزاق 9/4±23/13 است. نتایج حاکی از آن بود که رابطه معکوس معنی داری بین دو شاخص مورد اندازه گیری وجود داشت (337/0-=r و 001/0=P). نتیجه گیری: از دست دادن دندانها می تواند منجر به کاهش ظرفیت آنتی اکسیدانی بزاق و افزایش ریسک وقوع برخی بیماریها از جمله سرطان دهان گردد. واژه های کلیدی: ظرفیت آنتی اکسیدانی کل بزاق، بزاق کامل غیر تحریکی، از دست دادن دندان، سرطان دهان

Title: تعیین بهای تمام شده یک واحد عمل جراحی آپاندیس با استفاده از روش هزینه یابی بر مبنای فعالیت در بیمارستان شهید صدوقی یزد در سال 1390

http://tbj.ssu.ac.ir/article-1-1379-fa.html

مقدمه: هزینه یابی بر مبنای فعالیت، به عنوان ابزاری برای سنجش هزینه به منظور محاسبه دقیق و صحیح هزینه عملیات مورد استفاده قرار می گیرد.هدف پژوهش حاضر برآورد هزینه تمام شده ی یک واحد عمل جراحی آپاندیس در بیمارستان شهید صدوقی یزد با استفاده از روش هزینه یابی بر مبنای فعالیت در سال 1390 بوده است. روش بررسی: پژوهش حاضر از نوع پژوهش های کاربردی است که به شیوه توصیفی و به صورت مقطعی و گذشته نگر انجام شده است. داده های مورد نیاز از طریق مصاحبه با مسئولین بیمارستان و واحدهای ذیربط، مشاهده فعالیت های بخش جراحی و بررسی مدارک و دفاتر موجود دراین بخش وسایر واحدهای اداری و پشتیبانی جمع آوری گردیده است. سپس، برای برآورد سهم هزینه های سایر بخش ها در خدمات بخش جراحی، ابتدا مبنا های تسهییم تعیین سپس با استفاده از روش تسهیم دوطرفه (ریاضی)، هزینه ها برآورد وبا استفاده از رویکرد هزینه یابی بر مبنای فعالیت و با به کارگیری نرم افزار excel قیمت تمام شده یک واحد عمل جراحی آپاندیس محاسبه شده است. یافته ها : بر اساس یافته های پژوهش، بهای تمام شده یک واحد عمل جراحی آپاندیس 3,814,449 ریال محاسبه گردید .که هزینه دستمزد مستقیم با مبلغ 1099593 ریال 29 درصد از سهم کل هزینه ،هزینه دارو و لوازم مصرفی مستقیم با مبلغ 1162946ریال 30 درصد از سهم کل هزینه وهزینه سربار با مبلغ1551910ریال 41 درصد از سهم کل هزینه بهای تمام شده یک واحد عمل جراحی آپاندیس را تشکیل داده اند. نتیجه گیری: بر اساس یافته های پژوهش، می توان از طریق بهبود عملکرد، بخصوص اصلاح اقدامات مدیریت منابع انسانی و نیز استانداردسازی مصرف به منظور کاهش هزینه های مصرفی، قیمت تمام شده خدمات را کاهش داد.

Title: بررسی فراوانی انجام ماموگرافی و موانع انجام آن در بستگان نزدیک بیماران مبتلا به سرطان پستان در سیرجان

http://tbj.ssu.ac.ir/article-1-1380-fa.html

مقدمه: سرطان پستان شایع ترین نوع سرطان در بین زنان است و خطر بروز آن در زنانی که بستگان نزدیک آنها سابقه ابتلا را داشته باشند، بیشتر می باشد. لذا این مطالعه با هدف بررسی فراوانی انجام ماموگرافی و موانع انجام آن در بستگان نزدیک بیماران مبتلا به سرطان پستان انجام گردید. روش بررسی : جامعه مورد مطالعه شامل 65 زن از بستگان نزدیک ( اقوام درجه یک شامل مادر، دختر ، خواهر و اقوام درجه دو شامل خاله و عمه ) کلیه افراد مبتلا شده به سرطان پستان زیر سن یائسگی از سال1387 تا سال1391در شهر سیرجان بودند. اطلاعات توسط بخش موانع درک شده پرسشنامه چمپیون جمع آوری شده و با کمک نرم افزار SPSS مورد تجزیه و تحلیل قرار گرفت. یافته ها : براساس نتایج، 9/88 درصد افراد مورد بررسی، خانه دار، 6/84 درصد دارای بیمه و 4/75 درصد کمتر از 10 کیلومتر تا مرکز ماموگرافی فاصله داشتند، با این حال تنها 4/15 درصد زنان مورد مطالعه ماموگرافی انجام داده بودند. بین میانگین نمره موانع درک شده در زنانی که ماموگرافی انجام داده بودند ( 93/25 ) و زنانی که انجام نداده بودند ( 33/26 ) تفاوت معنی داری وجود نداشت. هزینه انجام ماموگرافی با میانگین رتبه ای 85/7، ترس از پیدا شدن سرطان پستان (72/6) و دردناک بودن ماموگرافی (97/5) بزرگترین موانع درک شده برای انجام ماموگرافی بودند. نتیجه گیری : انجام ماموگرافی از موانع درک شده در مورد آن تبعیت نمی کند و لازم است علت استقبال کم از این رفتار غربال گری را در زمینه های دیگر جستجو نمود. با این حال، هزینه های این رفتار و نیز مسئله ترس از پیدا شدن سرطان پستان و درد هنگام انجام ماموگرافی، مهمترین موانع گزارش شده ای است که توجه به آنها می تواند در ارتقاء این رفتار موثر باشد.

Title: شناسایی رویداد‌های استرس‌زا عمده سالمندان و رابطه آن‌ها با افسردگی و افت شناختی

http://tbj.ssu.ac.ir/article-1-1382-fa.html

مقدمه: بررسی های جهانی نشان می دهند که جمعیت سالمندی به دلیل ارتقاء سطح بهداشت در حال افزایش است. این در حالی است که این دوران چالش های بسیاری دارد و سالمندان مشکلات جسمانی و روانی زیادی را تجربه می کنند. جمعیت سالمندان در ایران نیز در حال افزایش است، بنابراین با توجه به اهمیت بهداشت روانی سالمندان، هدف پژوهش حاضر شناسایی رویداد‌های استرس‌زا عمده و رابطه آن‌ها با افسردگی و افت شناختی در سالمندان یزدی است. روش بررسی: پژوهش حاضر توصیفی از نوع همبستگی است. برای انتخاب گروه نمونه، از روش نمونه-گیری تصادفی خوشه‌ای چند‌مرحله‌ای استفاده شد، بدین شکل که از بین سالمندان شهر یزد 160 نفر انتخاب شدند. برای ارزیابی متغیر‌های پژوهش، از پرسشنامه وضعیت شناختی، رویدادهای استرس‌زا زندگی سالمندان (محقق ساخته) و مقیاس افسردگی سالمندان استفاده شد. داده ‌ها با روش‌های آماری تحلیل عاملی اکتشافی و آزمون همبستگی پیرسون بررسی شدند. یافته‌ها: نتایج پژوهش نشان دادند که رویدادهای مرتبط با مسائل مالی، مسائل اجتماعی، مسائل کاری، سلامت و مرگ و میر در سالمندان استرس‌زا بودند. هم‌چنین مسائل مالی، مسائل اجتماعی، مسائل کاری، سلامت و مرگ و میر با افسردگی رابطه داشته‌اند و مسائل اجتماعی با افت شناختی رابطه داشته است. نتیجه‌گیری: سالمندی زمان کاهش درآمد، کاهش روابط اجتماعی، از دست دادن نقش‌های اجتماعی، کاهش سلامتی و داغدیدگی است، که استرس ناشی از این عوامل با بروز علائم افسردگی در سالمندان رابطه معنی دار مثبت دارند. هم‌چنین استرس ناشی از کاهش روابط اجتماعی با بروز علائم افت شناختی رابطه معنی دار مثبت دارد.

Title: بررسی اثر مکمل کوآنزیم کیوتن بر فشار خون شریانی بیماران مبتلا به دیابت نوع دو

http://tbj.ssu.ac.ir/article-1-1383-fa.html

مقدمه : دیابت نوع دو یکی از علل مهم مرگ و میر در جهان محسوب می شود. ازجمله عوارض این بیماری فشار خون بالا می باشد. فشار خون بالا در افراد مبتلا به دیابت دو برابر بیشتر ار افراد معمولی است. امروزه در کنار درمان های دارویی در بیماری های اکسیداتیوی چون دیابت و فشار خون بالا ، از مکمل های آنتی اکسیدانی نظیر کوآنزیم کیوتن CoQ10)) استفاده می شود. این مطالعه با هدف بررسی تاثیرمکمل 0 CoQ1 بر فشار خون بیماران مبتلا به دیابت نوع دو طراحی شده است. روش بررسی : این مطالعه یک کارآزمایی بالینی تصادفی دو سوکور می باشد که با مشارکت 70 بیمار مبتلا به دیابت نوع دو تحت پوشش مرکز تحقیقات یزد در طول سال 1390 انجام گرفت. افراد به طور تصادفی به دو گروه CoQ10 و دارونما تقسیم شدند. هر دو گروه به مدت 12 هفته روزانه 2 کپسول 100 میلی گرمی محتوی CoQ10 یا دارونما مصرف کردند. فشار بیشینه، کمینه و فشار متوسط شریانی قبل و بعد از مطالعه اندازه گیری شد. یافته ها : در این مطالعه 62 نفر مشارکت کامل نمودند. میانگین فشار بیشینه، کمینه و فشار متوسط شریانی در گروه گیرنده CoQ10 بعد از دریافت مکمل نسبت به گروه شاهد کاهش معنی داری پیدا کرده بود (001/0( P<. میانگین فشار بیشینه در گروه دریافت کننده مکمل 65/12 میلیمتر جیوه نسبت به آغاز مطالعه کاهش یافت. این کاهش در فشار خون کمینه و متوسط فشار شریانی به ترتیب 37/11 و 8/11 میلی متر جیوه بود(001/0( P<. نتیجه گیری : مطالعه حاضر نشان می دهد مصرف مکمل CoQ10 در کاهش فشار خون بیماران مبتلا به دیابت نوع دو می تواند تا حدی موثر باشد. بنابر این با توجه به این اثرات و جهت جلوگیری از آسیب های ناشی از افزایش فشار خون مصرف این مکمل در بیماران دیابتی به ویژه بیماران دیابتی مبتلا به فشار خون بالا قابل توصیه است.

Title: بررسی مصرف سولفات روی برکاهش بروزاسهال ناشی از مصرف کو اموکسی کلاو در کودکان 6 تا 60 ماهه

http://tbj.ssu.ac.ir/article-1-1384-fa.html

مقدمه:اسهال یک عارضه نسبتا شایع بدنبال مصرف آنتی بیوتیک ها است. 25- 10% مصرف کنندگان کوآموکسی کلاو دچاراسهال می شوند. از طرفی روی، منجر به تقویت ایمنی ،مقاومت موکوزا و پوست به عفونت،رشدو توسعه سیستم عصبی می گردد. همچنین یک آنتی اکسیدان مهم و عاملی برای تثبیت غشای سلولی به شمار می رود. هدف از انجام این مطالعه بررسی تاثیر مصرف مکمل سولفات روی در پیشگیری ازبروز اسهال ناشی از مصرف کوآموکسی کلاو در کودکان 6 تا 60 ماهه می باشد. روش بررسی:دراین مطالعه که از نوع کار آزمایی بالینی دوسوکور بوده، 120 کودک 6 تا 60 ماهه در دو گروه 60نفره مورد(مصرف کننده آموکسی سیلین کلاوولانیک با روی) وشاهد(مصرف کننده آموکسی سیلین کلاوولانیک به تنهایی) مورد بررسی قرارگرفتند. یافته ها : نتایج نشان داد که 26 نفر از 120 کودک مورد مطالعه (%7/21) بدنبال مصرف آموکسی سیلین کلاوولانیک دچار اسهال شدند.درگروه مورد 18 نفراز59 نفر(%5/30) و در گروه شاهد 8 نفراز 61 نفر( % 1/ 13) دچار اسهال شدند(018/0 P =). نتیجه گیری: اسهال بدنبال مصرف آموکسی سیلین کلاوولانیک درکودکان شایع بوده و مصرف سولفات روی نه تنهادر پیشگیری این موضوع تاثیرندارد بلکه ممکن است فراوانی اسهال را افزایش دهد. .
